# Reply to Dr. Max Greenbaum

**Published:** 1895-02

**Authors:** Edward C. Kirk


### Domestic Correspondence.

### REPLY TO DR. MAX GREENBAUM.

To the Editor:

Dear Sir,—I find in recent issues of your valued journal two articles by Max Greenbaum, D.D.S., of Philadelphia, which purport to be a reply to a paper of mine on "Coagulants in the Treatment of the Pulp-Chambers and Canals," published in the *Dental Cosmos* for March, this year. I have spent some time and careful study in trying to discover just what my critic is endeavoring to establish. He has evidently demolished, to his entire satisfaction, several propositions which he has himself set up for the purpose, but that they have any existence in my paper, or anywhere outside of his own imagination, I challenge him, or any one else, to show. He has assumed that my research was intended to demonstrate the superiority of certain antiseptic coagulants over others in the treatment of root-canals. In support of this statement I quote from his paper in the International Dental Journal for November, page 706, as follows: "The position taken by Dr. Kirk in his recent article has, we believe, been shown to be far from convincing, *and far from its purpose to disprove the assertion that such coagulants as carbolic acid or zinc chloride are detrimental when employed in canal work.*" I never had any such purpose, and distinctly disavowed it in the article he criticises. In support of which assertion I quote from my article the following statement of the problem which I proposed to investigate:

"The objection which is made to the use of coagulants by those who condemn them in this connection is, as nearly as I have been able to ascertain, based upon the assumption—for I regard it as such—that when introduced into a pulp-chamber their action is to superficially seal the dentinal tubuli by forming an impenetrable coagulum with the orificial end of their albuminoid contents. This is believed to produce the two-fold result of preventing the further entrance of the coagulant or any other antiseptic, and also of hermetically enclosing within the tubular structure of the dentine a certain quantity of non-sterilized or infected organic matter, which may, by its decomposition, act subsequently as an irritant to the pericemental membrane. If the major premise of this assumption be a correct statement of the actual conditions, then we must admit the corollaries as deduced from it; but in so doing we necessarily admit that our best-known and most generally used antiseptics in the treatment of pulp-canals—viz., carbolic acid, zinc chloride, and corrosive sublimate—do not penetrate the dentine structure, and consequently sterilize only a superficial film of organic matter lining the pulp-chamber and canals. The long clinical record in favor of the substances named would seem to indicate that their action in the pulp-canal is something more than a superficial action, and that their penetrating power is not self-limited because of the coagulating property which is common to each. It is my purpose to call attention to what I believe to be a serious error in this reasoning, which has led to the somewhat general condemnation of coagulants in pulp-canal treatment, and to present the bases for my belief that the coagulating property is not *per se* a sufficient reason for their disuse; or, stated otherwise, if they are unsuited for the purpose of pulp-canal treatment, it must be for other reasons than that they are coagulants. . . .

"I do not wish to be understood as favoring the indiscriminate use of these substances, for they possess individual characteristic properties and effects which may rightfully exclude their usage under certain conditions. What I wish to emphasize is that their coagulating property cannot be scientifically or clinically urged as an objection against their proper use."

The problem which I had under consideration was a problem of physics, not one of therapeutics, and it would seem reasonable that any one of average intelligence and a fair capacity for understanding the meaning of English words would have gathered that idea from reading the article in question. Dr. Greenbaum announces as a new fact, presumably the result of his own investigations, that "We found different coagulants act in different degrees as relates to power of coagulation, the different coagula showing a marked difference in structure." This observation deserves respect because of its antiquity. It is not quite so haggard as the Wandering Jew, but it is at least venerable on account of its age. In this connection I would suggest that if he will pursue his investigations a little further, he will find that carbolic acid, zinc chloride, and corrosive sublimate will have their coagulating power modified in degree proportionate to the strength of solution in which they are used.

By following this up for a while he will be able to produce coagula exactly analogous to those which he gets from campho-phénique (which depends for its coagulating action on the carbolic acid, which is its most prominent antiseptic constituent), oil of cloves, or oil of cinnamon. In neither of his critical effusions has the writer touched upon the only point of issue in my article,—viz., the self-limiting character of coagulated albumen,—excepting when he says, "and we may find, furthermore, that albumen coagulated solidly, such as results from the action of carbolic acid or zinc chloride, admits of no extended penetration of a second application, unless such application remained in contact in excessive quantity with the coagulum for two days or more. In dental work this is not admissible." This is really to the point, and would be interesting if it were true. The writer does not seem to be quite sure of his position, inasmuch as his statement is couched in the protective covering of the subjunctive mood. Now, if he will read carefully once more the article which he has criticised, he will find recorded there experiments intended to determine this very point. Let me, however, state for his further enlightenment that solid coagula, three-sixteenths of an inch thick, made with albumen and zinc chloride in saturated solution, permitted the passage of mercuric chloride solution in forty-five minutes. Dr. Greenbaum is quite at liberty to draw his own conclusions as to the applicability of these data to practical dental therapeutics; in fact, it was for gaining more accurate knowledge in this direction that the physical problem of osmosis through coagula was investigated by me. My critic, instead of showing errors of observation or deduction in my paper, has wasted effort and valuable space in a dental journal in attempting to explode propositions of his own manufacture. His energy would, I think, have been better expended in a careful study of the paper which he criticises. It might have helped him to a better understanding of a subject which, if his treatment of it is an indication, he is most profoundly in need of. His own position in this matter he characterizes as "scientific," and, by implication at least, that set forth by his opponent as "unscientific." I submit, Mr. Editor, that his recent writings furnish evidence of the fact that he either has only a very vague idea, or none at all, of the content of the term scientific, or that his language does not express his ideas. In support of the former proposition I offer as proof his article, "The One Material," International Dental Journal, September, 1894, and of the latter, the closing sentence in the November issue, as follows: "The statements of laws of any scientific knowledge are the statements of laws of scientific dentistry, and thus it advances to the position of a true science." Just what form of paranoia has attacked my critic I am unable to diagnose, but its two most prominent symptoms seem to be an acute logorrhœa accompanied with intellectual tenesmus. Logorrhœa you will not find in the dictionary, but it is derived from *logos*, word; the remainder of the derivation will be self-evident.

EDWARD C. KIRK.

November 7, 1894.

